# Rapid Diagnostic Testing for Response to the Monkeypox Outbreak — Laboratory Response Network, United States, May 17–June 30, 2022

**DOI:** 10.15585/mmwr.mm7128e1

**Published:** 2022-07-15

**Authors:** Tricia A. Aden, Patricia Blevins, Shannon Whitman York, Stacy Rager, Devika Balachandran, Christina L. Hutson, David Lowe, Chris N. Mangal, Tyler Wolford, Audrey Matheny, Whitni Davidson, Kimberly Wilkins, Rachael Cook, Rebecca M. Roulo, Michael K. White, LaShondra Berman, Janna Murray, John Laurance, Drew Francis, Nicole M. Green, Ricardo A. Berumen, Anthony Gonzalez, Stacy Evans, Meghan Hudziec, Diane Noel, Michael Adjei, Gregory Hovan, Phil Lee, Lisa Tate, Remedios B. Gose, Robert Voermans, Jennifer Crew, Phillip R. Adam, Danielle Haydel, Salimatu Lukula, Nick Matluk, Sandip Shah, Joshua Featherston, Daphne Ware, Denise Pettit, Emily McCutchen, Edward Acheampong, Erin Buttery, Andrew Gorzalski, Michael Perry, Randal Fowler, Robert B. Lee, Robert Nickla, Richard Huard, Amanda Moore, Katie Jones, Renee Johnson, Erin Swaney, Juan Jaramillo, Cynthia Reinoso Webb, Brandon Guin, Janine Yost, Annette Atkinson, Latoya Griffin-Thomas, Jessica Chenette, Jessica Gant, Alana Sterkel, Harjinder K. Ghuman, James Lute, Sandra C. Smole, Vaneet Arora, Courtney K. Demontigny, Meilan Bielby, Evelyn Geeter, Kimberly A. M. Newman, Mark Glazier, Whitney Lutkemeier, Megan Nelson, Raymond Martinez, Jasmine Chaitram, Margaret A. Honein, Julie M. Villanueva

**Affiliations:** ^1^Division of Preparedness and Emerging Infections, National Center for Emerging and Zoonotic Infectious Diseases, CDC; ^2^Deloitte Consulting, LLP, Atlanta, Georgia; ^3^Division of High-Consequence Pathogens and Pathology, National Center for Emerging and Zoonotic Infectious Disease, CDC; ^4^Association of Public Health Laboratories, Silver Spring, Maryland; ^5^St. John Group, Atlanta, Georgia; ^6^Alaska State Public Health Laboratory, Fairbanks, Alaska; ^7^Arizona State Public Health Laboratory, Phoenix, Arizona; ^8^Los Angeles County Public Health Laboratory, Los Angeles, California; ^9^California Department of Public Health Laboratory, Sacramento, California; ^10^Sacramento County Public Health Laboratory, Sacramento, California; ^11^San Luis Obispo County Public Health Department, San Luis Obispo, California; ^12^Colorado Department of Public Health & Environment; ^13^Dr. Katherine A. Kelley Public Health Laboratory, Connecticut Department of Public Health; ^14^District of Columbia Public Health Laboratory; ^15^Delaware Public Health Laboratory, Smyrna, Delaware; ^16^Florida Bureau of Public Health Laboratories-Jacksonville, Jacksonville, Florida; ^17^Florida Bureau of Public Health Laboratories-Tampa, Tampa, Florida; ^18^Hawaii State Laboratories Division, Pearl City, Hawaii; ^19^Idaho Bureau of Laboratories, Boise, Idaho; ^20^Chicago Department of Public Health Laboratory, Chicago, Illinois; ^21^Kansas Health & Environmental Laboratories, Topeka, Kansas; ^22^Louisiana Public Health Laboratory, Baton Rouge, Louisiana; ^23^Maryland Laboratories Administration, Baltimore, Maryland; ^24^Maine State Health and Environmental Testing Laboratory, Augusta, Maine; ^25^Michigan Public Health Laboratory, Lansing, Michigan; ^26^Missouri State Public Health Laboratory, Jefferson City, Missouri; ^27^Mississippi Public Health Laboratory, Jackson, Mississippi; ^28^North Carolina State Laboratory of Public Health, Raleigh, North Carolina; ^29^Nebraska Public Health Laboratory, Omaha, Nebraska; ^30^New Jersey Division of Public Health & Environmental Laboratories; ^31^Southern Nevada Public Health Laboratory, Reno, Nevada; ^32^Nevada State Public Health Laboratory; ^33^Wadsworth Center, New York State Department of Health; ^34^New York City Department of Health and Mental Hygiene Bureau of Laboratories, New York; ^35^Ohio Department of Health Laboratories, Reynoldsburg, Ohio; ^36^Oregon State Public Health Laboratory, Hillsboro, Oregon; ^37^Rhode Island State Health Laboratories, Providence, Rhode Island; ^38^South Carolina Bureau of Laboratories, Columbia, South Carolina; ^39^Knoxville Regional Public Health Laboratory, Knoxville, Tennessee; ^40^Tennessee Department of Health: Laboratory Services; ^41^Texas Department of State Health Services; ^42^Dallas County Health and Human Services, Dallas, Texas; ^43^Texas Tech University Bioterrorism Response Laboratory, Lubbock, Texas; ^44^San Antonio Metro Health District Laboratory, San Antonio, Texas; ^45^Public Health Laboratory of East Texas, Tyler, Texas; ^46^Unified Utah State Laboratories: Public Health, Taylorsville, Utah; ^47^Virginia Division of Consolidated Laboratory Services, Richmond, Virginia; ^48^Vermont Department of Health Laboratory, Colchester, Vermont; ^49^Washington Public Health Laboratories, Shoreline, Washington; ^50^Wisconsin State Laboratory of Hygiene, Madison, Wisconsin; ^51^Georgia Public Health Laboratory, Decatur, Georgia; ^52^Pennsylvania Bureau of Laboratories, Exton, Pennsylvania; ^53^Massachusetts State Public Health Laboratory, Jamaica Plain, Massachusetts; ^54^Kentucky Division of Laboratory Service, Frankfort, Kentucky; ^55^Minnesota Public Health Laboratory Division, St. Paul, Minnesota; ^56^Houston Public Health Laboratory, Houston, Texas; ^57^Alabama Bureau of Clinical Laboratories, Montgomery, Alabama; ^58^Montana Laboratory Services Bureau, Helena, Montana; ^59^Indiana Public Health Laboratory, Indianapolis, Indiana; ^60^South Dakota Public Health Laboratory, Pierre, South Dakota; ^61^State Hygienic Laboratory of the University of Iowa, Coralville, Iowa; ^62^Corpus Christi - Nueces County Health Department Laboratory, Corpus Christi, Texas; ^63^Division of Laboratory Systems, Center for Surveillance, Epidemiology, and Laboratory Services, CDC.

As part of public health preparedness for infectious disease threats, CDC collaborates with other U.S. public health officials to ensure that the Laboratory Response Network (LRN) has diagnostic tools to detect *Orthopoxviruses*, the genus that includes *Variola virus*, the causative agent of smallpox. LRN is a network of state and local public health, federal, U.S. Department of Defense (DOD), veterinary, food, and environmental testing laboratories. CDC developed, and the Food and Drug Administration (FDA) granted 510(k) clearance* for the Non-variola *Orthopoxvirus* Real-time PCR Primer and Probe Set (non-variola Orthopoxvirus [NVO] assay), a polymerase chain reaction (PCR) diagnostic test to detect NVO. On May 17, 2022, CDC was contacted by the Massachusetts Department of Public Health (DPH) regarding a suspected case of monkeypox, a disease caused by the *Orthopoxvirus Monkeypox virus*. Specimens were collected and tested by the Massachusetts DPH public health laboratory with LRN testing capability using the NVO assay. Nationwide, 68 LRN laboratories had capacity to test approximately 8,000 NVO tests per week during June. During May 17–June 30, LRN laboratories tested 2,009 specimens from suspected monkeypox cases. Among those, 730 (36.3%) specimens from 395 patients were positive for NVO. NVO-positive specimens from 159 persons were confirmed by CDC to be monkeypox; final characterization is pending for 236. Prompt identification of persons with infection allowed rapid response to the outbreak, including isolation and treatment of patients, administration of vaccines, and other public health action. To further facilitate access to testing and increase convenience for providers and patients by using existing provider-laboratory relationships, CDC and LRN are supporting five large commercial laboratories with a national footprint (Aegis Science, LabCorp, Mayo Clinic Laboratories, Quest Diagnostics, and Sonic Healthcare) to establish NVO testing capacity of 10,000 specimens per week per laboratory. On July 6, 2022, the first commercial laboratory began accepting specimens for NVO testing based on clinician orders.

LRN was established in 1999^†^ as a partnership among CDC, the Federal Bureau of Investigation, and the Association of Public Health Laboratories, with the goal of ensuring a laboratory infrastructure across the United States that can respond quickly and effectively to biothreats, chemical threats, and emerging infectious diseases (*1*). LRN provides the framework to rapidly distribute laboratory diagnostic tests, standardized reagents, and standard operating procedures, and to train laboratory personnel, report laboratory test results, and provide critical communication during routine and emergency responses. LRN includes approximately 110 U.S. laboratories, primarily state and local public health and DOD laboratories, as well as veterinary, food, and environmental testing laboratories. LRN laboratories are required to participate in proficiency testing exercises to ensure competency for laboratory test methods distributed to the network.

To effectively respond to a potential *Orthopoxvirus* outbreak, CDC subject matter experts worked with LRN to design, develop, and validate an assay to detect NVOs, such as *Vaccinia, Cowpox, Monkeypox,* and *Ectromelia* viruses, if suspected cases were identified. The NVO assay first received 510(k) clearance by FDA in 2005 and was cleared again in 2018 to update the labeling and use of reagents. The NVO assay does not differentiate *Monkeypox virus* from other O*rthopoxviruses*. NVOs are not endemic in the United States; however, the NVO assay has been used to detect cases of *Vaccinia virus* infection associated with vaccination and two imported cases of monkeypox from travelers in 2021 (*2*).

CDC recommends that for each patient, clinicians collect two specimens, each from multiple lesions, preferably from different locations on the body and from lesions with differing appearances (*3*). The CDC *Monkeypox virus* testing algorithm includes NVO testing, and if results are positive for *Orthopoxvirus*, further characterization testing at CDC (*4*). A subset of specimens was characterized at CDC by a *Monkeypox virus* specific real-time PCR assay and genetic sequencing.^§^ The median LRN laboratory testing turnaround time was calculated from the time of specimen receipt by LRN testing laboratories to arrival of NVO test results at CDC. Testing capacity was estimated and reported by LRN laboratories. This report describes NVO testing by LRN during May 17–June 30, 2022. This investigation was reviewed by CDC and conducted consistent with applicable federal law and CDC policy.^¶^

As of June 10, 68 U.S. LRN laboratories, located in 47 states and the District of Columbia, had implemented the NVO assay updated in 2018 and tested specimens from patients with probable monkeypox cases. These laboratories reported an estimated total testing capacity of 8,000 specimens per week. LRN laboratories reported that capacity of NVO testing laboratories was limited by reagent availability and the requirement for manual DNA extraction. To increase testing throughput and build capacity, the NVO assay was rapidly updated to include additional controls, automated extraction, and real-time PCR instrumentation in collaboration with FDA; the updated assay received 510(k) clearance on June 10, 2022. As of June 30, 2022, 78 LRN laboratories had implemented the NVO assay and have reported a total testing capacity of 24,000 specimens per week with implementation of substantial operational changes such as adding extra shifts, reassigning personnel, and shifting testing priorities based on laboratory emergency response plans.

During May 17–June 30, a total of 2,009 specimens were tested in LRN laboratories (Table); 730 (36.3%) specimens from 395 persons across 31 jurisdictions (including 29 states, District of Columbia, and Puerto Rico) were confirmed positive for *Orthopoxvirus* using the NVO assay. One positive specimen from each patient (159) was sent to CDC and further characterized as *Monkeypox virus* belonging to the West African clade; as of June 30, 236 confirmed *Orthopoxvirus* cases were pending characterization. The median LRN laboratory testing turnaround time was 30.7 hours for all results (Table).

**TABLE:** Number of specimens* tested for non-variola *Orthopoxvirus* and testing turnaround times, by week — Laboratory Response Network, United States, May 17–June 30, 2022

Date range, 2022	No. of specimens tested	No. (%) positive for NVO^†^	Median turnaround time from specimen receipt to CDC report, hrs	
			All	Positive
May 17–23	25	16 (64.0)	34.1	34.1
May 24–30	57	3 (5.3)	28.2	23.7
May 31–Jun 6	164	38 (23.2)	30.0	19.2
Jun 7–13	334	80 (24.0)	28.4	27.4
Jun 14–20	350	138 (39.4)	25.2	25.3
Jun 21–27	647	237 (36.6)	37.9	44.7
Jun 28–30	432	218 (50.4)	30.9	37.0
**Total, May 17–Jun 30**	**2,009**	**730 (36.3)**	**—**	**—**
**Cumulative median**	**—**	**—**	**30.7**	**30.2**

**Abbreviation:** NVO = non-variola *Orthopoxvirus.*

*Number of specimens exceeds number of cases because some persons had multiple specimens collected for testing.

^†^All paired specimens sent to CDC were confirmed as *Monkeypox virus.*

Although LRN laboratories provide initial recognition and detection of emerging infectious diseases, rapid expansion of nationwide testing capacity was indicated for this outbreak. Therefore, CDC obtained 510(k) clearance from FDA on June 23 to enable CDC to provide the NVO assay to five large commercial laboratories under a licensing agreement that included CDC training and test verification before the start of testing. This expansion of testing provides additional test capacity and electronic laboratory reporting to public health authorities, makes testing more accessible, and streamlines diagnostic testing for multiple, possible infections. When fully operational, these five national commercial laboratories are anticipated to increase weekly testing capacity nationwide by approximately 10,000 specimens per laboratory.

## Discussion

CDC and LRN have collaborated with public health partners to prepare for *Orthopoxvirus* outbreaks, enabling rapid public health response through the development and expansion of testing capacity and medical countermeasures to prevent the spread of disease. Laboratory preparedness efforts included NVO test validation, FDA 510(k) clearance, distribution, and verification of diagnostic tests to detect NVO. This response highlights the importance of preparedness against emerging infectious diseases and the need to further strengthen and expand LRN to include other partners to enhance testing capability and increase surge testing capacity.

Because monkeypox disease has been rare in the United States, CDC's NVO assay is the only FDA 510(k)–cleared assay to detect NVO; at the onset of this outbreak, use of the assay was limited to LRN laboratories. The 510(k) clearance facilitated rapid testing and detection of a rare, high-risk, and emerging pathogen by LRN laboratories by maintaining competency and biosafety practices, results reporting, and collaborating with public health authorities, all essential to the initial national response.

CDC recommends that U.S. health care providers be alert for patients who have rash illnesses consistent with monkeypox (*5*) and include NVO testing as part of their clinical workup. Clinicians who suspect a case of monkeypox can contact their local or state health department** for specimen submission guidance. A rapid turnaround time for test results is critical to quickly initiate public health action to better control the spread of monkeypox disease. Treatment is the same for all NVO infections; thus, a positive test result for an *Orthopoxvirus* using the NVO assay is immediately actionable, leading to the use of antiorthopoxviral treatment, if warranted, and allowing public health authorities to initiate isolation, contact tracing, monitoring, investigation, and postexposure prophylaxis of exposed contacts (*5*). In addition, if monkeypox is suspected based on clinical signs and symptoms, clinicians can initiate treatment, advise patients to isolate while awaiting test results, and take measures to prevent further transmission, like limiting close contact with others or avoiding the sharing of potential contaminated items. Tecovirimat (TPOXX) can also be prescribed as treatment for people with monkeypox, and two vaccines, JYNNEOS and ACAM2000 (*6*) can be provided to close contacts as postexposure prophylaxis. By the end of June 2022, <10% of the available nationwide LRN NVO testing capacity had been used. Despite the high capacity, some clinicians and patients reported challenges navigating public health testing procedures, including acquiring public health approvals for testing. Expansion to five commercial laboratories starting the week of July 5 should make testing more accessible, increase convenience for providers and patients by both using existing provider-laboratory relationships and eliminating the need for prior public health approval, and further augment national capacity. Expanded testing access via both LRN and commercial laboratories provides the opportunity to identify all cases of *Orthopoxvirus* to enhance monitoring and response to the outbreak.

**Summary**

**What is already known on this topic?**

The Laboratory Response Network (LRN) includes U.S. laboratories validated to perform the non-variola *Orthopoxvirus* (NVO) assay.

**What is added by this report?**

During May 17–June 30, 2022, LRN laboratories tested 2,009 specimens from patients with suspected monkeypox. Among these, 730 (36%) specimens from 395 patients were positive for NVO. Specimens from 159 persons with NVO-positive results were confirmed by CDC to be monkeypox; confirmatory testing is pending for 236. LRN laboratories have increased testing capacity from 8,000 per week in June because of NVO assay updates.

**What are the implications for public health practice?**

LRN laboratories’ rapid results enable prompt patient treatment and prevention of further transmission. Expansion of testing to five large national laboratories will increase ease of access to testing.

## Footnotes

*
https://www.fda.gov/medical-devices/device-approvals-denials-and-clearances/510k-clearances

†
https://emergency.cdc.gov/lrn/usmap.asp

§
https://www.biorxiv.org/content/10.1101/2022.06.10.495526v1

¶ 45 C.F.R. part 46, 21 C.F.R. part 56; 42 U.S.C. Sect. 241(d); 5 U.S.C. Sect. 552a; 44 U.S.C. Sect. 3501 et seq.

**
https://cdn.ymaws.com/www.cste.org/resource/resmgr/poc/epioncall_update1622.pdf
